# A Targeted Radiotheranostic Agent for Glioblastoma: [^64^Cu]Cu-NOTA-TP-c(RGDfK)

**DOI:** 10.3390/brainsci15080844

**Published:** 2025-08-07

**Authors:** Alireza Mirzaei, Samia Ait-Mohand, Prenitha Mercy Ignatius Arokia Doss, Étienne Rousseau, Brigitte Guérin

**Affiliations:** 1 Department of Medical Imaging and Radiation Sciences, Faculty of Medicine and Health Sciences, Université de Sherbrooke, Sherbrooke, QC J1H 5N4, Canada; alireza.mirzaei@usherbrooke.ca (A.M.); samia.ait-mohand@usherbrooke.ca (S.A.-M.); prenitha.mercy.ignatius.arokia.doss@usherbrooke.ca (P.M.I.A.D.); etienne.rousseau@usherbrooke.ca (É.R.); 2 Sherbrooke Molecular Imaging Center (CIMS), Centre de Recherche du Centre Hospitalier Universitaire de Sherbrooke (CRCHUS), 3001, 12e Avenue Nord, Sherbrooke, QC J1H 5N4, Canada; 3 Institut de Recherche sur le Cancer de l’Université de Sherbrooke (IRCUS), Université de Sherbrooke, Sherbrooke, QC J1H 5N4, Canada

**Keywords:** glioblastoma multiforme (GBM), radiotheranostics, integrin α_v_β_3_ targeting, copper-64, terpyridine-platinum complex

## Abstract

Glioblastoma multiforme (GBM) remains one of the most aggressive and treatment-resistant brain tumors, with poor prognosis and limited therapeutic options. **Background/Objectives**: Integrin α_v_β_3_, a cell surface receptor overexpressed in GBM, specifically binds to cyclic arginine-glycine-aspartate-D-phenylalanine-lysine (c(RGDfK)) motif, making it a valuable target for tumor-specific delivery and PET imaging. This study explores a novel radiotheranostic agent, [^64^Cu]Cu-NOTA-TP-c(RGDfK), which combines the imaging and therapeutic capabilities of copper-64 (^64^Cu) and the cytotoxic activity of a terpyridine-platinum (TP) complex, conjugated to c(RGDfK). **Methods**: A robust protocol was developed for the small-scale preparation of NOTA-TP-c(RGDfK). Comparative cellular studies were conducted using U87 MG glioblastoma (GBM) cells and SVG p12 human astrocytes to evaluate the performance of [^64^Cu]Cu-NOTA-TP-c(RGDfK) relative to [^64^Cu]Cu-NOTA-c(RGDfK), [^64^Cu]Cu-NOTA-TP, ^nat^Cu-NOTA-TP-c(RGDfK), cisplatin, and temozolomide. **Results**: ^64^Cu-radiolabeling of NOTA-TP-c(RGDfK) was achieved with >99% radiochemical purity, and competition assays confirmed high binding affinity to integrin α_v_β_3_ (IC_50_ = 16 ± 8 nM). Cellular uptake, internalization, and retention studies demonstrated significantly higher accumulation of [^64^Cu]Cu-NOTA-TP-c(RGDfK) in U87 MG cells compared to control compounds, with 38.8 ± 1.8% uptake and 28.0 ± 1.0% internalization at 24 h. Nuclear localization (6.0 ± 0.5%) and stable intracellular retention further support its therapeutic potential for inducing localized DNA damage. Importantly, [^64^Cu]Cu-NOTA-TP-c(RGDfK) exhibited the highest cytotoxicity in U87 MG cells (IC_50_ = 10 ± 2 nM at 48 h), while maintaining minimal toxicity in normal SVG p12 astrocytes. **Conclusions**: These results highlight [^64^Cu]Cu-NOTA-TP-c(RGDfK) as a promising targeted radiotheranostic agent for GBM, warranting further preclinical development

## 1. Introduction

Glioblastoma multiforme (GBM) is an aggressive and malignant brain tumor originating from astrocytes, a type of glial cell [1,2]. It accounts for approximately 16% of all brain tumors [3] and is characterized by its invasive growth, rapid progression, and resistance to conventional treatments [4]. The prognosis for GBM patients remains poor, with an average survival period of 12 to 15 months following diagnosis [5].

The standard treatment approach for GBM includes a combination of surgery, radiation therapy, and the chemotherapy agent temozolomide (TMZ), an oral alkylating agent that alters the function and structure of DNA of cancer cells [6]. However, due to the infiltrative nature of GBM, complete surgical removal of the tumor is often challenging, leading to frequent recurrence [7]. Despite the clear unmet medical need, progress in developing new treatments for GBM has been minimal over the past several decades.

Integrins, particularly the α_v_β_3_ integrin, play a significant role in the invasive nature and progression of GBM [8]. These heterodimeric transmembrane proteins, composed of α and β subunits, are involved in key processes like cell proliferation, migration, invasion, and angiogenesis, making them attractive therapeutic targets [9]. Among the various integrin subtypes, α_v_β_3_ is highly expressed in high-grade brain tumors and exhibits specific binding to the Arg-Gly-Asp (RGD) sequence found in extracellular matrix proteins such as fibronectin and vitronectin [10]. While linear RGD peptides have been explored for targeting integrins, their clinical application is limited due to their low binding affinity, lack of specificity, and rapid degradation by serum proteases. To address these limitations, cyclic arginine-glycine-aspartate-D-phenylalanine-lysine c(RGDfK) have been developed [11]. This cyclic peptide, that incorporates a D-amino acid residue (D-Phe), offers significant advantages due to its enhanced binding affinity, selectivity, and stability, making it well-suited for therapeutic delivery [12]. A variety of ^18^F- and ^68^Ga-labeled c(RGDfK) peptides have been explored for PET imaging of GBM, demonstrating strong diagnostic potential in both preclinical and clinical settings [13,14,15,16]. Compared to monomeric peptides, multimeric RGD-based radiopharmaceuticals offer longer integrin-specific tumor retention in vivo and improved pharmacokinetics [17]. An alternative strategy to enhance cancer cell accumulation and improve the pharmacokinetic profile of RGD peptides could involve increasing their affinity for plasma proteins—particularly serum albumin—to extend circulation time and promote tumor accumulation through the enhanced permeability and retention (EPR) effect and albumin-mediated transport [18].

In recent decades, the combination of multiple cancer treatment modalities—such as chemotherapy, radiotherapy, immunotherapy, and targeted therapy—has improved therapeutic efficacy while minimizing toxicity to surrounding healthy tissues. For instance, platinum (Pt)-based chemotherapeutic agents have been combined with radiation therapy, producing effects ranging from supra-additive to sub-additive across various cell lines [19,20,21,22]. Maximal effects have been observed in vivo when irradiation is applied 4 h and 48 h after cisplatin treatment, coinciding with peak platinum binding to tumor cell DNA [23]. Clinical studies show that daily cisplatin administration for 5 days before radiation increases treatment efficacy by 35% compared to radiotherapy alone [24]. Pt-based drugs exert cytotoxicity by binding to DNA, forming adducts that destabilize the DNA double helix, disrupt replication and mitosis, and ultimately lead to cell death [21]. However, the effectiveness of these drugs is limited by severe side effects such as nephrotoxicity, neurotoxicity, and ototoxicity, as well as the potential for acquired or intrinsic resistance to Pt-based chemotherapy [25,26]. To address these challenges, various families of platinum square planar complexes, such as terpyridine platinum (TP), have been synthesized. TP exhibits a high affinity for intercalating with guanine-rich motifs on DNA, particularly G-quadruplexes, making them valuable therapeutic targets [27,28,29,30]. The human genome contains approximately 350,000 guanine-rich motifs capable of forming G-quadruplexes, primarily located on telomeres and oncogene promoters, particularly in the upstream region of the c-MYC gene promoter [31,32]. Targeting these DNA regions with TP-based chemotherapeutic agents can enhance G-quadruplex stability, resulting in displacement of the shelterin complex from telomeres [33]. This disruption triggers a DNA damage response and telomere attrition, ultimately inducing cell senescence and apoptosis [31].

To address resistance to platinum-based drugs, we developed two ^64^Cu-labeled NOTA-TP compounds as chemoradiotheranostic (CRT) agents specifically targeting colorectal cancer cells [34,35]. In these studies, the ^64^Cu isotope was chosen as an effective dual-purpose radionuclide for PET imaging and radiotherapy due to its favorable emission profile—positron emission (0.653 MeV, 17.8%), electron capture (43.1%), β^−^ decay (0.579 MeV, 38.4%)—and half-life of 12.7 h. Our results showed that [^64^Cu]Cu-NOTA-TP were stable, more cytotoxic (17,500–55,000×) and more selective for cancer cells than other non-radioactive platinum compounds, including oxaliplatin [34,35]. Moreover, we have demonstrated that these CRT agents exhibit dual binding to albumin and DNA, enabling non-invasive tumor visualization by PET imaging while concurrently delivering therapeutic effects [34,35,36]. Finally, although the first-generation NOTA-TP compounds combined with ^64^Cu showed a strong supra-additive and selective cytotoxic effect [28], their molecular design may still have been suboptimal.

**Figure 1:**
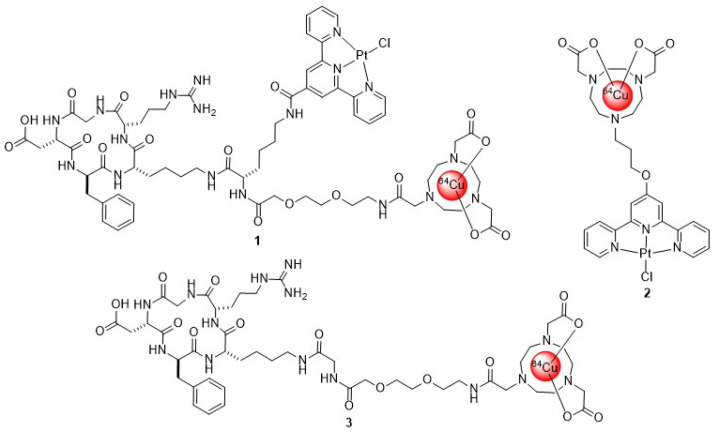
The chemical structure of [^64^Cu]Cu-NOTA-TP-c(RGDfK) **1**, [^64^Cu]Cu-NOTA-TP **2** and [^64^Cu]Cu-NOTA-c(RGDfK) **3**.

Building on recent advancements, we propose that a compound combining ^64^Cu with NOTA-TP and c(RGDfK) peptide could significantly improve therapeutic outcomes in GBM. This novel agent is designed to leverage the selective binding affinity of c(RGDfK) for the overexpressed α_v_β_3_ integrin receptors in GBM, thereby improving targeted drug delivery and therapeutic efficacy. The objective of this study is to rigorously assess the targeting capability and therapeutic potential of [^64^Cu]Cu-NOTA-TP-c(RGDfK) **1** (Figure 1) through comprehensive in vitro assays. Comparative analyses with the monomeric analogs, [^64^Cu]Cu-NOTA-TP **2** and [^64^Cu]Cu-NOTA-c(RGDfK) **3** (Figure 1), will further elucidate the specificity and efficacy of this compound against GBM cells. In this context, we evaluated the radiotherapeutic potential of [^64^Cu]Cu-NOTA-TP-c(RGDfK) using in vitro models, including U87 malignant glioblastoma (MG) cells and SVG p12 human normal astrocyte cell lines.

## 2. Materials and Methods

### 2.1. Chemistry

#### 2.1.1. General

All chemicals and solvents were used as received from the suppliers unless otherwise specified. 2-Chlorotrityl chloride resin was purchased from Chem-Impex International Inc. (Wood Dale, IL, USA). Fluorenylmethyloxycarbonyl- (Fmoc)-protected amino acids were sourced from EMD NovaBiochem (Gibbstown, NJ, USA) or Chem-Impex International Inc. 2,2′,2″-(1,4,7-triazacyclononane-1,4,7-triyl)triacetic acid (NOTA) derivative was obtained from CheMatech (Dijon, France). Acetonitrile (CH_3_CN), dichloromethane (DCM), *N*,*N*-dimethylformamide (DMF), and methanol (MeOH) were supplied by Fisher Scientific (Ottawa, ON, Canada). DMF was dried over 4 Å molecular sieves for at least one week and filtered prior to use to eliminate trace amines. All instruments were calibrated and maintained according to standard quality control procedures. Mass spectra were acquired using an API 3000 LC/MS/MS (Applied Biosystems/MDS SCIEX, Concord, ON, Canada), a Waters/Alliance HT 2795 system with a Waters 2996 PDA and a Waters Micromass ZQ detector, an API 2000, and an ESI-Q-Tof (MAXIS). The NOTA-c(RGDfK) derivatives were purified using a Biotage HPFC SP4 Flash Purification System with a C18 column. Analytical HPLC was conducted on an Agilent 1200 system (Agilent Technologies, Mississauga, ON, Canada) equipped with a Zorbax Eclipse XDB C18 reversed-phase column (4.6 × 250 mm, 5 μm) and a 1200 series diode array UV-Vis detector. The method used was as follows: flow rate of 1 mL/min; 0–23 min: gradient from 0 to 76.6% CH_3_CN with 0.05% trifluoroacetic acid (TFA) in H_2_O with 0.05% TFA; 23–24 min: 100% CH_3_CN; 24–30 min: return to 0% CH_3_CN.

^64^Cu was produced using a TR-19 or TR-24 cyclotron (ACSI) via the ^64^Ni(p,n)^64^Cu nuclear reaction, employing an enriched ^64^Ni target electroplated onto a rhodium disc. The resulting ^64^Cu-chloride (^64^CuCl_2_) was isolated following the method described by McCarthy et al. and subsequently converted to ^64^Cu-acetate (^64^Cu(OAc)_2_) by dissolving it in 0.1 M ammonium acetate buffer (pH 5.5) [34].

#### 2.1.2. Synthesis of NOTA-c(RGDfK) **4** and NOTA-TP-c(RGDfK) **5**

**Scheme 1:**
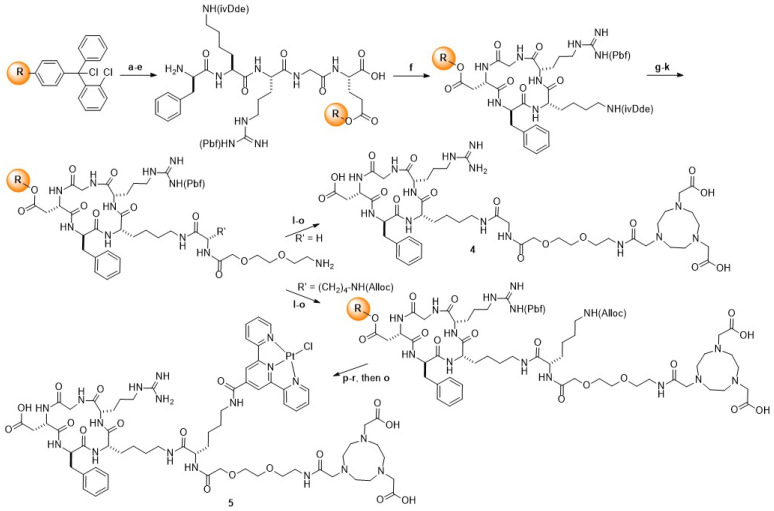
General scheme for the synthesis of NOTA-c(RGDfK) **4** and NOTA-TP-c(RGDfK) **5**. Reagents and conditions: (a) Fmoc-Asp(OAll)-OH, DIPEA, DCM; (b) Piperidine, DMF; (c) Fmoc-AA-OH, HATU, DIPEA, DMF; (d) PhSiH_3_, Pd(PPh_3_)_4_; (e) Piperidine, DMF; (f) PyBoP, HOBt, DIPEA, DMF; (g) 2% NH_2_NH_2_, DMF; (h) Fmoc-Gly-OH, HATU, DIPEA, (R′=H) or Fmoc-Lys(Alloc)-OH, HATU, DIPEA (R′=(CH_2_)_4_-NH(Alloc); (i) Piperidine, DMF; (j) Fmoc-PEG-OH, HATU, DIPEA; (k) Piperidine, DMF; (l) bromoacetic acid, EDC, DCM; (m) TACN, DCM; (n) *tert*butyl bromoacetate, DIPEA, *N*-methylpyrrolidone; (o) TFA, H_2_O, TIPS; (p) Pd(PPh_3_)_4_, morpholine, DCM, (q) 2,2′:6′,2″-terpyridine-4′-carboxylic acid, HATU, DIPEA, DMF; (r) K_2_PtCl_4_, DMF, H_2_O. R = 2-chlorotrityl-chloride resin.

c(RGDfK) peptides **4** and **5** were synthesized via solid-phase peptide synthesis protocols using commercially available 2-chlorotrityl chloride resin (loading capacity: 0.8 mmol/g), Scheme 1. HATU was employed as the coupling reagent, and Fmoc-amino acids were used. Prior to synthesis, the resin was swollen 30 min in DCM [37]. The synthesis began with the attachment of Fmoc–Asp(OAll)–OH (2 equivalents (equiv)) to the resin in the presence of *N*,*N*-diisopropylethylamine (DIPEA, 4 equiv) in DCM for 4 h. Fmoc deprotection was carried out by treating the resin with 20% piperidine in DMF for three cycles of 10 min each. The subsequent four amino acids—Fmoc–Gly–OH, Fmoc–Arg(Pbf)–OH, Fmoc–Lys(ivDde)–OH, and Fmoc–D-Phe–OH—were coupled sequentially using 5 equiv of each Fmoc-amino acid, activated in situ with HATU (5 equiv) and DIPEA (10 equiv) in DMF, with each coupling allowed to proceed for 3 h. Coupling efficiency at each step was monitored using the Kaiser test. After each reaction, the resin was thoroughly washed with DMF, DCM and MeOH. Before the final Fmoc deprotection, the selective removal of the allyl protecting group from the α-carboxyl of Asp was achieved by treating the peptidyl resin with phenylsilane (PhSiH_3_, 24 equiv) and tetrakis(triphenylphosphine)palladium(0) (Pd(PPh_3_)_4_, 0.25 equiv) in DCM for 3 h. This was followed by washes using a DIPEA:DMF solution (5:95) and a solution of sodium diethyldithiocarbamate in DMF to remove palladium residues.

The head-to-tail cyclization of the linear peptide was performed using benzotriazol-1-yloxytripyrrolidinophosphonium hexafluorophosphate (PyBOP, 1.5 equiv), hydroxybenzotriazole (HOBt, 1.5 equiv), and DIPEA (2 equiv) in DMF under millimolar concentration for 5 h. The 1-(4,4-dimethyl-2,6-dioxocyclohex-1-ylidene)-3-methylbutyl (ivDde) protecting group on the lysine side chain was selectively and efficiently removed by treating the resin-bound peptide with 2% hydrazine monohydrate in DMF (10 × 2 min). Fmoc–Gly–OH (for peptide **4**) and Fmoc–Lys(Alloc)-OH (for peptide **5**) were then coupled to the ε-amino group of lysine using HATU (5 equiv) and DIPEA (10 equiv) in DMF for 3 h. The Fmoc group was then removed with 20% piperidine in DMF (2 × 10 min), followed by coupling of Fmoc–PEG–OH under the same conditions (HATU, DIPEA in DMF); a final Fmoc deprotection step was performed. Once orthogonal deprotection was achieved, the NOTA peptide was prepared following bromoacetylation, triazacyclononane (TACN) introduction, and alkylation with *tert*-butyl bromoacetate procedures as previously described [38].

NOTA-c(RGDfK) **4**. The resin was cleaved with a cocktail of TFA:H_2_O:triisopropylsilane (TIPS) (95:2.5:2.5) for 3 h. The resin was removed by filtration and washed with TFA. Combined filtrates were added dropwise to cold diethyl ether. The precipitated crude peptide was centrifuged, and the ether solution was decanted. Crude peptide **4** was purified by flash chromatography on a Biotage SP4 system, using a C18 cartridge. The product fractions were pooled and lyophilized to obtain the desired compound with 28% yield. Purity of the peptide was verified by HPLC and accounted for 99%; the identity was confirmed by API 3000 LC/MS/MS. HPLC: Retention time (R_t_) = 11.47 min; ESI-MS: calcd: 1092.6, found, 1094.0 [M+1], 547 [M/2], (Figure S1).

NOTA-TP-c(RGDfK) **5**. The Alloc protecting group on the lysine side chain of the resin-bound peptide was selectively removed by treatment with Pd(PPh_3_)_4_ (0.25 equiv) and morpholine (30 equiv) in DCM for 3 h. The resin was then thoroughly washed with a DIPEA–DMF solution (5:95, *v*/*v*), followed by a solution of sodium diethyldithiocarbamate in DMF to ensure complete removal of palladium residues. Subsequently, 2,2′:6′,2″-terpyridine-4′-carboxylic acid [39] was coupled to the deprotected amine on resin using HATU and DIPEA in DMF for 3 h. Final washes were performed with DMF, DCM, and MeOH. Platination of the TP ligand was carried out by treating the resin-bound peptide with 10 equiv of K_2_PtCl_4_ in a H_2_O:DMF (1:9, *v*/*v*) mixture, followed by gentle agitation for 18 h. The resin was washed thoroughly with water and DMF, followed by additional washes with DMF alone, DCM and MeOH. Cleavage of the resin-bound peptide was performed using a cleavage cocktail TFA:H_2_O:TIPS (95:2.5:2.5) for 3 h at room temperature. The resin was then removed by filtration and washed with TFA. Cold diethyl ether was added to the filtrate to precipitate the crude peptide. The precipitate was collected by centrifugation, and the ether supernatant was decanted. The resulting crude peptide **5** was purified by flash chromatography using a Biotage SP4 system equipped with a C18 reverse-phase cartridge. The product fractions were pooled and lyophilized to obtain the desired compound with a poor yield of 2–3%. Peptide purity was assessed by analytical reverse-phase HPLC, and its identity was confirmed by mass. HPLC: R_t_ = 12.76 min; ESI-MS: calcd: 1652.4, found, 1653.7 [M+1], 826.9 [M/2], Figure S2.

#### 2.1.3. Synthesis of NOTA-TP

This compound was prepared as described previously [35].

#### 2.1.4. Synthesis of ^nat^Cu-NOTA-c(RGDfK) and ^nat^Cu-NOTA-TP-c(RGDfK)

^nat^Cu-NOTA-c(RGDfK) and ^nat^Cu-NOTA-TP-c(RGDfK) were prepared by dissolving the c(RGDfK) peptides **4** and **5** in a minimal amount of DMSO, followed by the addition of a slight excess (1.1 equiv) of trace-metal-grade copper(II) acetate (Cu(OAc)_2_) in 0.1 M ammonium acetate buffer at pH 5.5.

^nat^Cu-NOTA-c(RGDfK) Upon stirring at room temperature for 20 min, the solution turned from white to green. The compound was purified on C18 sept Pak and eluted with ethanol, and isolated as a pale green of the desired complex with a yield of 90% and a purity of 99%. ESI-MS: calcd: 1154.9; found, 1196.87 [M+2Na], 599.0 [M/2+Na], Figure S3.

^nat^Cu-NOTA-TP-c(RGDfK). Upon stirring at room temperature for 1 h, the initially orange solution gradually turned green. After this period, a green precipitate was formed, collected by filtration, and thoroughly washed with water then dried. The compound was obtained as a green solid with a 75% yield. ESI-MS: calcd 1736.3; found, 1735.0 [M-1], 867.8 [M/2], 579.0 [M/3], 434.5 [M/4], Figure S4.

#### 2.1.5. Radiolabeling of NOTA-Conjugates

Radiolabeling was carried out by incubating 5–10 nmol of NOTA-TP-c(RGDfK), NOTA-c(RGDfK) or NOTA-TP with 500–1200 MBq of ^64^Cu(OAc)_2_ in 500 μL of 0.1 M ammonium acetate buffer (pH 7.2) at room temperature for 20 min. The labeling efficiency was confirmed by radio-instant thin layer chromatography (iTLC), using C18 plates and 0.1 M sodium citrate buffer (pH 5.5) as the mobile phase. Radiodetection was performed with an Instant Imager system (BioScan, Washington, D.C., USA). Under these conditions, free ^64^Cu migrates with the solvent front, whereas the radiolabeled complex [^64^Cu]Cu–NOTA–TP-c(RGDfK) remains at the origin, Figure S5A,B.

### 2.2. Stability Studies

#### Plasma Stability and Protein Binding

Plasma stability of [^64^Cu]Cu-NOTA-TP-c(RGDfK) was evaluated by incubating the radiolabeled compound (100–200 MBq in 250 μL PBS) with 250 μL of mouse plasma at 37 °C for up to 48 h. To precipitate plasma proteins, the samples were treated twice with ethanol (1:1, *v*/*v*), vortexed for 1 min, and centrifuged at 7000 rpm for 10 min. The soluble fraction was separated by ultracentrifugation, and radioactivity was measured. The resulting supernatant was analyzed by radio-TLC on C18 plates. Free ^64^Cu(OAc)_2_ and [^64^Cu]Cu–NOTA–TP-c(RGDfK) were used as standards. Radio-TLC was developed using 0.1 M sodium citrate buffer (pH 5.5), and radiodetection was performed with an Instant Imager system (BioScan, Washington, DC, USA) (Figure S5C). The supernatant fraction was then analyzed using the same methodology described above.

To assess protein binding, radioactivity in both the supernatant and precipitated protein fractions was quantified using a dose calibrator to determine the percentage of protein-bound radioactivity.

### 2.3. Cellular Assays

#### 2.3.1. Cell Culture

U87 MG and SVG p12 cell lines obtained from ATCC were cultured in Dulbecco’s modified Eagle medium (DMEM) and Eagle’s minimal essential medium (EMEM), respectively. These culture media were supplemented with 10% FBS, 2 mM L-glutamine, and a mix of penicillin (100 UI/mL), amphothericin B (250 µg/mL) and streptomycin (100 µg/mL). Cells were incubated at 37 °C in a humidified environment with 5% CO_2_. In all cellular assays, ^64^CuTP-based compounds were formulated in 0.7 mM BSA [36].

#### 2.3.2. Competition Assay

U87 MG cells (1 × 10^5^) were seeded into 24-well polylysine-coated Cellbind plates. After allowing the cells to grow for two days to reach 70–90% confluency, the cell culture medium was replaced with 400 µL of reaction medium composed of DMEM, 2% HEPES, 1% penicillin/streptomycin, and 20 g/L bovine serum albumin. Subsequently, 50 µL of [^64^Cu]Cu-NOTA-c(RGDfK) (7.5 nM, 0.07–0.09 MBq/well) was added to each well along with 50 µL of inhibitors, ranging in concentration from 10^−3^ to 10^−13^ M. The inhibitors used were ^nat^Cu-NOTA-c(RGDfK) and ^nat^Cu-NOTA-TP-c(RGDfK). Following a 1 h incubation period, the medium was aspirated, and the cells were washed once with 500 µL of cold PBS. The cells were then trypsinized and collected for radioactive measurements using gamma counter. The assays were performed in triplicate, with independent productions of [^64^Cu]Cu-NOTA-c(RGDfK) and separate cell seedings for each trial. IC_50_ values were calculated using the GraphPad Prism 9 software, employing a one-site fit logIC50 model.

#### 2.3.3. Cellular Uptake, Internalization, and Efflux Kinetics of the ^64^Cu-NOTA Conjugates

U87 MG (1 × 10^5^ per well) were seeded in corning 24-well plates and cultured in DMEM with 10% FBS at 37 °C. At 80% confluency, the cells were then incubated with [^64^Cu]Cu-NOTA-TP-c(RGDfK) and their controls [^64^Cu]Cu-NOTA-TP and [^64^Cu]Cu-NOTA-c(RGDfK) at a sub-lethal concentration and incubated for 1 to 48 h, to measure cellular uptake and internalization. For uptake assay, cells were washed with PBS to collect surface-bound and internalized activity, and for internalization assay, glycine-HCl wash was performed to remove surface bound activity. Moreover, cells were harvested, counted, and radioactivity was measured using a gamma counter. Results were expressed as percentage of total activity (%) in counts per min (CPM)/10^6^ cells. For efflux studies, U87 MG cells were incubated with the complexes for 1 h, then washed with PBS, and replaced with fresh medium. The retained activity was measured over 0 to 24 h after washing.

#### 2.3.4. Comparative Subcellular Localization of [^64^Cu]Cu-NOTA-TP-c(RGDfK) in U87 MG Versus SVG p12 Cells

The nucleus and cytoplasm of U87 MG and SVG p12 cells were extracted following established procedures as previously reported [34]. U87 MG and SVG p12 cells were treated with 1 MBq of [^64^Cu]Cu-NOTA-TP-c(RGDfK) for 24, 48, and 72 h. After incubation, cells were washed and incubated on ice with CSK buffer (0.5% Triton X-100, 300 mM sucrose, 100 mM NaCl, 1 mM EDTA, 2 mM MgCl_2_, and 10 mM HEPES, pH 6.8) for 2 min. After 5 min centrifugation, supernatant containing cytoplasmic extract was collected and transferred in labeled tubes. The resulting pellets were incubated on ice for 2 min with CSK buffer without Triton X-100 to extract the nuclear fraction. The buffer containing the nuclear fraction was collected into separate tubes. The radioactivity in both cytoplasmic and nuclear fractions was measured using a gamma counter (HIDEX Automatic Gamma Counter 2014).

#### 2.3.5. Cytotoxicity Assay

The cytotoxicity of the complexes was evaluated using the PrestoBlue (Thermo Fisher Scientific), a resazurin-based metabolic assay known for its speed and high sensitivity in assessing cell viability and cytotoxicity [40]. In this procedure, U87 MG (2 × 10^4^ cells per well) were seeded in BD falcon transparent 96-well plates and incubated for 24 h. Subsequently, the cells were treated with [^64^Cu]Cu-NOTA-TP-c(RGDfK) and their controls ([^64^Cu]Cu-NOTA-TP, [^64^Cu]Cu-NOTA-c(RGDfK), cisplatin and TMZ) at concentrations ranging from 0 to 300 nM for 24, 48, and 72 h. After removing the media, the cells were washed with warm PBS and exposed to PrestoBlue reagent for 30 min. Changes in cell viability were measured through fluorescence (λ_ex_ = 570 nm: λ_em_ = 610 nm). Dose–response curves were generated by plotting the concentration of the drugs versus fluorescence values, allowing the calculation of median effective concentration (EC_50_) values.

### 2.4. Data Analysis

All experiments were performed in triplicate, and the results were expressed as mean values ± S.D. A *p*-value of less than 0.05 was significant (two-tailed independent Student’s *t*-test). Calculations were performed with GraphPad Prism 7.0 (GraphPad Software, Inc., La Jolla, CA, USA).

## 3. Results

### 3.1. Synthesis and Radiolabeling

NOTA-c(RGDfK) **4** and NOTA-TP-c(RGDfK) **5** were synthesized on solid phase using standard Fmoc-based peptide chemistry, yielding overall product yields of 28% and 2–3%, respectively (Scheme 1, Figures S1 and S2). The preparation of the corresponding c(RGDfK) analogs labeled with natural copper (^nat^Cu) proceeded quantitatively (Figures S3 and S4). ^nat^Cu-NOTA-TP-c(RGDfK) shows limited solubility in aqueous media, requiring a co-solvent system of 10% DMSO in saline to fully dissolve the ^nat^Cu-complex. Stability studies performed by mass spectrometry at physiological pH (7.0) revealed no detectable release of copper or platinum over 72 h, underscoring the high stability of the complex under physiological conditions. All compounds are >95% pure by HPLC. Radiolabeling with ^64^Cu(OAc)_2_ was carried out efficiently, yielding a quantitative radiochemical conversion (>99%) (Figure S5A,B) and an apparent molar activity of 102–190 MBq/nmol for [^64^Cu]Cu-NOTA-c(RGDfK) and 98–120 MBq/nmol for [^64^Cu]Cu-NOTA-TP-c(RGDfK) and [^64^Cu]Cu-NOTA-TP.

### 3.2. Stability Studies

Preliminary protein binding evaluation demonstrated that 52% of the total ^64^Cu from [^64^Cu]Cu-NOTA-TP-c(RGDfK) remained bound to plasma proteins after 48 h of incubation at 37 °C in mouse plasma. Analysis of the supernatant by radio-TLC (Figure S5C) revealed no detectable free ^64^Cu, supporting the high stability of the radiolabeled complex in biological fluids.

### 3.3. Cellular Assays

The inhibitory concentration 50 (IC_50_) values for ^nat^Cu-NOTA-c(RGDfK) and ^nat^Cu-NOTA-TP-c(RGDfK) were determined on the human malignant glioblastoma cell line U87 MG through competition with [^64^Cu]Cu-NOTA-c(RGDfK), as shown in Table 1 (Figure S6). The IC_50_ of ^nat^Cu-NOTA-c(RGDfK) was 10.6 ± 3.2 nM, indicating a strong affinity for the integrin α_v_β_3_-expressing U87 MG cells (entry 1). ^nat^Cu-NOTA-TP-c(RGDfK) exhibited a slightly lower affinity with an IC_50_ of 16.2 ± 6.1 nM (entry 2). The value was not significantly different from that determined in competition with ^nat^Cu-NOTA-c(RGDfK) (*p* = 0.25).

**Figure 2:**
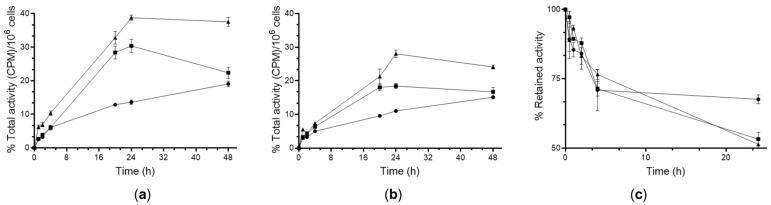
Cellular uptake (**a**), internalization (**b**) and efflux (**c**) of [^64^Cu]Cu-NOTA-TP-c(RGDfK) (black triangle) in comparison with [^64^Cu]Cu-NOTA-TP (black square) and [^64^Cu]Cu-NOTA-c(RGDfK) (black circle) in human U87 MG cell line expressing integrin-α_v_β_3_. Cellular uptake and internalization are presented as % of total activity (CPM)/10^6^ cells. Efflux is expressed as the percentage of retained activity at t = 0, when the radioactive media was removed.

We assessed the cellular uptake kinetics of ^64^Cu-conjugates over a 48 h period at 37 °C in the U87 MG cell line. Our results showed that all three tested ^64^Cu-conjugates reached peak accumulation at 24 h, which was sustained at a plateau through 48 h (Figure 2a, Table S1). Notably, among the tested compounds, [^64^Cu]Cu-NOTA-TP-c(RGDfK) demonstrated the highest cellular accumulation. At the 24 h time point, the uptake of [^64^Cu]Cu-NOTA-TP-c(RGDfK) was approximately 1.3 times higher than that of [^64^Cu]Cu-NOTA-TP and 2.7 times higher than [^64^Cu]Cu-NOTA-c(RGDfK), as shown in Figure 2a.

The kinetics of total cell-internalized fractions of ^64^Cu-conjugates were evaluated after washing U87 MG cells with glycine-HCl (Figure 2b, Table S2). The internalized fraction of [^64^Cu]Cu-NOTA-TP-c(RGDfK) increased markedly from 5.5 ± 0.8% CPM/10^6^ cells at 1 h to a peak of 28.0 ± 1.0% CPM/10^6^ cells at 24 h, consistent with previous findings for ^64^Cu-labeled TP agents [34,35]. At this time point, [^64^Cu]Cu-NOTA-TP-c(RGDfK) showed over 1.5-fold higher internalization compared to [^64^Cu]Cu-NOTA-TP, and more than 2.4-fold higher than [^64^Cu]Cu-NOTA-c(RGDfK).

The efflux assays were conducted to quantify the activity of ^64^Cu-conjugates pumped out of U87 MG cells (Figure 2c, Table S3). After a 1 h incubation with the ^64^Cu-conjugates, the compounds were removed, and efflux kinetics were monitored over a 24 h period. Approximately 20–30% of the ^64^Cu-conjugates were rapidly eliminated within the first 4 h, followed by a slower efflux rate, with over 50% of the compounds still retained in U87 MG cells at 24 h. The efflux rate of [^64^Cu]Cu-NOTA-c(RGDfK) is slower than that of the two ^64^Cu-TP conjugates.

**Figure 3:**
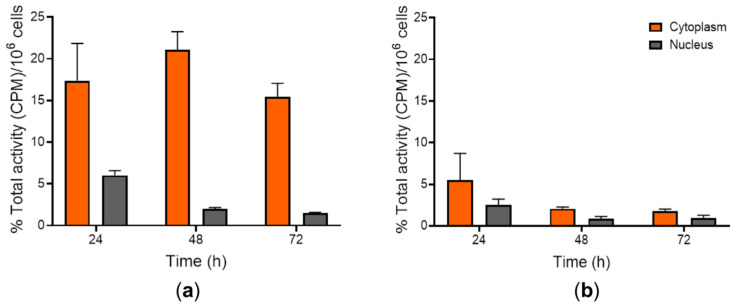
Subcellular accumulation of [^64^Cu]Cu-NOTA-TP-c(RGDfK) in (**a**) U87 MG cells and (**b**) SVG p12 normal astrocyte as function of incubation time. Data are presented as the % of total activity (CPM)/10^6^ cells.

The subcellular distribution of [^64^Cu]Cu-NOTA-TP-c(RGDfK) was analyzed in U87 MG cancer cells and SVG p12 normal glial cells over 24, 48, and 72 h (Figure 3). In U87 MG cells, nuclear uptake of [^64^Cu]Cu-NOTA-TP-c(RGDfK) progressively decreased over time, reaching a peak of 6.0 ± 0.5% at 24 h. Cytoplasmic distribution fluctuated, measuring 17.4 ± 1.5% at 24 h, 21.0 ± 0.7% at 48 h, and then 15.4 ± 0.5% at 72 h. Across the same time points, nuclear uptake of [^64^Cu]Cu-NOTA-TP-c(RGDfK) in SVG p12 cells remained consistently lower, starting 2.5 ± 0.8% at 24 h, declining to 0.9 ± 0.2% at 48 h, and further dropping to 0.9 ± 0.3% at 72 h. Cytoplasmic accumulation in these cells followed a similar downward trend.

Cell viability was assessed using PrestoBlue, a resazurin-based method that evaluates mitochondrial activity in both U87 MG glioblastoma cells and SVG p12 normal glial cells following treatment with various concentrations of ^64^Cu-conjugates. ^nat^Cu-NOTA-TP-c(RGDfK), cisplatin and TMZ were included as control chemotherapeutic agents. The EC_50_ values, presented in Table 2, were derived from dose–response curves for each compound (Figure S7).

^nat^Cu-NOTA-TP-c(RGDfK) exhibited cytotoxicity at the micromolar (μM) range in U87 MG cells, with a moderate time-dependent increase in potency (Table 2, entry 1). However, this non-radioactive conjugate showed only modest selectivity over normal SVG p12 cells. [^64^Cu]Cu-NOTA-TP-c(RGDfK) showed nanomolar potency in U87 MG cells at all time points tested, representing an improvement of approximately 2500-fold at 24 h compared to its nonradioactive analogue, suggesting an additive effect between ^64^Cu and the NOTA-TP-c(RGDfK) (Table 2, entry 1 vs. entry 2). [^64^Cu]Cu-NOTA-TP-c(RGDfK) also exhibited higher selectivity than ^nat^Cu-NOTA-TP-c(RGDfK) toward U87 MG cells compared to SVG p12 cells, especially at early time points (Table 2, entry 1 vs. entry 2. A similar enhancement in cancer cell cytotoxicity and selectivity has also been observed with ^64^Cu-labeled TP agents. [34,35].

The monomeric counterparts [^64^Cu]Cu-NOTA-TP and [^64^Cu]Cu-NOTA-c(RGDfK) maintained comparable nanomolar potency at 24 and 48 h post-incubation but exhibited slightly lower cytotoxicity for U87 MG cells at all evaluated time points compared to [^64^Cu]Cu-NOTA-TP-c(RGDfK) (Table 2, entries 3 and 4 vs. entry 2). These ^64^Cu-conjugates are more selective towards U87 cell line compared to SVG p12 cell line. Cisplatin, used as a chemotherapeutic control drug, showed EC_50_ values comparable to those of ^nat^Cu-NOTA-TP-c(RGDfK) in U87 MG cells (Table 2, entry 5 vs. entry 1), but demonstrated greater potency in SVG p12 normal glial cells. TMZ—the clinical reference drug for GBM—was the least potent agent in U87 MG cells and displayed greater cytotoxicity toward normal glial cells at all evaluated time points (Table 2, entry 6).

## 4. Discussion

Radiolabeled c(RGDfK) peptides with high affinity and specificity toward tumor-associated α_v_β_3_ integrins have shown great promise in cancer diagnosis and therapy [17]. In this study, we evaluated [^64^Cu]Cu-NOTA-TP-c(RGDfK), a novel CRT agent designed to improve specificity, binding to plasma proteins and cytotoxicity for GBM cancer cells.

NOTA-c(RGDfK) **4** and NOTA-TP-c(RGDfK) **5** were obtained with overall yields of 28% and 2–3%, respectively. The low overall yield of 3% of compound **5** can be attributed to several compounding factors. First, the synthesis involves head-to-tail cyclization of the RGDfK peptide, a strategy known to be more challenging and typically associated with yields in the range of 10–25% due to entropic and steric constraints. Following cyclization, the peptide was further functionalized with a terpyridine ligand and platinum, which significantly increased the molecular weight and hydrophobicity of the final compound. These modifications introduced poor aqueous solubility and likely led to substantial losses during purification and handling, particularly during HPLC purification and lyophilization, where precipitation and adsorption to surfaces can occur. Additionally, coordination with platinum may contribute to product aggregation or formation of multiple species, further complicating purification and reducing the apparent isolated yield.

All conjugates were obtained with a purity exceeding 95%, but the synthesis of c(RGDfK) derivatives remains complex and results in a very low overall yield. ^64^Cu-radiolabeling of the CRT agents was achieved with high radiochemical purity and excellent molar activity. As shown in our previous studies, high molar activity is crucial for ^64^Cu-labeled TP derivatives to achieve cytotoxic effects in the low nanomolar range [34,35]. The current studies show that [^64^Cu]Cu-NOTA-TP-c(RGDfK) has an excellent stability over 48 h. It is noteworthy to mention that [^64^Cu]Cu-NOTA-TP-c(RGDfK) retained its ability to bind bovine serum albumin (52%), although to a lesser extent than the [^64^Cu]Cu-NOTA-TP conjugates (94–96%) [34,36].

The RGD-derivatizes retained nanomolar affinity for α_v_β_3_ integrin, as confirmed in competitive binding assays against U87 MG cells. The modest decrease in binding affinity of ^nat^Cu-NOTA-TP-c(RGDfK) relative to ^nat^Cu-NOTA-c(RGDfK) may be attributed to steric hindrance introduced by the TP moiety. Cellular uptake studies revealed that [^64^Cu]Cu-NOTA-TP-c(RGDfK) exhibited significantly higher accumulation in U87 MG cells compared to both [^64^Cu]Cu-NOTA-TP (*p* = 0.024) and [^64^Cu]Cu-NOTA-c(RGDfK) (*p* = 0.0001), reaching 38.8 ± 1.8% at 24 h.

The high internalization rate of [^64^Cu]Cu-NOTA-TP-c(RGDfK) may result from a combined effect of dual receptor engagement—namely, integrin-mediated endocytosis [41] and potentially albumin receptor-mediated transcytosis [42]. Albumin is known to be internalized by cancer cells through the gp60 receptor, which is overexpressed at the surface of many cancer cells, including U87 MG cells [43]. The enhanced selectivity of [^64^Cu]Cu-NOTA-TP-c(RGDfK) toward cancer cells may be attributed to its dual-targeting internalization mechanism, as both α_v_β_3_ integrin and gp60 receptors are overexpressed in U87 MG cells compared to normal glial cells [13,14,15,16,41,43].

Efflux studies indicated prolonged intracellular retention of all three ^64^Cu-labeled CRT agents, with more than 50% of the activity remaining after 24 h. Notably, [^64^Cu]Cu-NOTA-c(RGDfK) exhibited the highest retention in U87 MG cells. It has been reported that RGD are internalized and retained in melanoma cells and specifically interacts with survivin, a known cell-cycle and survival-regulator highly expressed in melanoma cells [44]. Survivin is also highly expressed in human U87 MG cancer cells [45], which may contribute to the elevated intracellular retention of c(RGDfK)—a hypothesis that warrants further validation.

These results demonstrate a distinct subcellular distribution pattern of [^64^Cu]Cu-NOTA-TP-c(RGDfK) between cancerous U87 MG cells and normal SVG p12 cells, with the ^64^Cu-CRT agent showing the highest accumulation at 24 h in both the cytoplasm and nucleus of U87 MG cells. This nuclear localization is particularly relevant given that ^64^Cu emits low-energy Auger electrons with a short range (<10 μm), which are highly cytotoxic when deposited near DNA.

The TP moiety, known to non-covalently interact with DNA G-quadruplex structures in telomeric and oncogenic promoter regions, may serve as a targeting vector for these radiation-sensitive domains. This interaction could potentially enhance the induction of double-stranded DNA breaks and promote cell death. Cytotoxicity assays confirmed the therapeutic potency of the [^64^Cu]Cu-NOTA-TP-c(RGDfK) with IC_50_ values in the low nanomolar range for U87 MG cells. This CRT agent exhibited over 11,000-fold and 7000-fold greater potency compared to cisplatin and TMZ, respectively. Furthermore, [^64^Cu]Cu-NOTA-TP-c(RGDfK) exhibited significantly lower toxicity in normal astrocyte cells (SVG p12), suggesting a wide therapeutic window.

Several ^64^Cu-RGD-based PET tracers have been designed for integrin αvβ3 imaging in glioma, such as ^64^Cu-DOTA-RGD tetramers [46] and PEGylated ^64^Cu-RGD peptides [47]. These tracers have shown favorable tumor-targeting characteristics and pharmacokinetics in U87MG human glioblastoma xenograft model [46,47]. Although these ^64^Cu-based agents are primarily diagnostic agents and lack cytotoxic payloads, they provide valuable data supporting the possibility of integrin-targeted uptake in GBM, which can inform the design of future theranostic strategies. In contrast, [^64^Cu]Cu-NOTA-TP-c(RGDfK) combines αvβ3 integrin targeting, a DNA-interacting platinum moiety, and the theranostic properties of copper-64 within a single construct.

Based on its dual targeting mechanism and cytotoxic profile, [^64^Cu]Cu-NOTA-TP-c(RGDfK) might have potential as a theranostic agent for both diagnosis and treatment of glioblastoma. [^64^Cu]Cu-NOTA-TP-c(RGDfK) targets αvβ3 integrin, a receptor expressed in high-grade gliomas, which could allow non-invasive PET imaging to assess integrin expression status, patient stratification, and treatment planning. Furthermore, the internalization and nuclear localization of the complex in cancer cells—mediated by both integrin- and albumin-related uptake—could enable localized radiotoxicity through the short-range Auger electron emission of ^64^Cu. This property may be relevant for addressing infiltrative tumor cells that are not removed during surgical resection.

## 5. Conclusions

This proof-of-concept study supports the potential use of [^64^Cu]Cu-NOTA-TP-c(RGDfK) as a novel CRT agent for GBM treatment. Taken together, [^64^Cu]Cu-NOTA-TP-c(RGDfK) emerges as a promising CRT candidate, integrating selective α_v_β_3_ integrin targeting, albumin-mediated binding enhancement, and the radiotherapeutic properties of ^64^Cu into a single agent—offering a rational strategy to improve both cancer cell specificity and cytoxicity. Given that the delivery of therapeutic agents across the blood–brain barrier remains a major challenge in glioblastoma, additional in vivo investigations—particularly in orthotopic models—will be essential to evaluate therapeutic efficacy of [^64^Cu]Cu-NOTA-TP-c(RGDfK). Future studies are also warranted to evaluate biodistribution, conduct dosimetric analysis, and elucidate the contributions of albumin-mediated transport and G-quadruplex DNA interactions to overall therapeutic outcomes.

## Figures and Tables

**Table 1:** Affinities of c(RGDfK) ligands for α_v_β_3_ integrins on human GBM (U87 MG) cell.

Entry	c(RGDfK) Ligand ^a^	IC_50_ on U87 MG (nM)
1	^nat^Cu-NOTA-c(RGDfK)	10.6 ± 3.2
2	^nat^Cu-NOTA-TP-c(RGDfK)	16.2 ± 6.1

^a^ In competition to [^64^Cu]Cu-NOTA-c(RGDfK).

**Table 2:** EC_50_ values (μM) of therapeutic agents for both human cancer (U87 MG) and normal glial cells (SVG p12).

Entry	Compound	24 h		48 h		72 h	
		U87 MG	SVG p12	U87 MG	SVG p12	U87 MG	SVG p12
1	^nat^Cu-NOTA-TP-c(RGDfK)	42 ± 4	75 ± 7	27 ± 4	40 ± 7	13 ± 7	35 ± 3
2	[^64^Cu]Cu-NOTA-TP-c(RGDfK)	0.017 ± 0.002	>0.300	0.010 ± 0.002	0.089 ± 0.008	0.007 ± 0.002	0.040 ± 0.005
3	[^64^Cu]Cu-NOTA-TP	0.026 ± 0.002	>0.300	0.021 ± 0.001	0.081 ± 0.003	0.020 ± 0.003	0.041 ± 0.004
4	[^64^Cu]Cu-NOTA-c(RGDfK)	0.025 ± 0.007	>0.300	0.032 ± 0.009	>0.200	0.045 ± 0.002	0.118 ± 0.008
5	Cisplatin	90 ± 8	35 ± 4	25 ± 6	15 ± 7	20 ± 5	15 ± 5
6	Temozolomide	800 ± 11	80 ± 10	580 ± 8	76 ± 7	575 ± 6	72 ± 5

Mean ± SD are shown; *n* = 3.

## Data Availability

All data generated or analyzed during this study are either included in this published article and its Supplementary Materials files or are available from the corresponding author upon reasonable request.

## Supplementary Materials

The following supporting information can be downloaded at: https://www.mdpi.com/article/10.3390/brainsci15080844/s1, Figure S1. LC/MS/MS spectrum and HPLC chromatogram of NOTA-c(RGDfK) **4**; Figure S2. ESI-MS spectrum and HPLC chromatogram of NOTA-TP-c(RGDfK) **5**; Figure S3. ESI-MS spectrum of natCu-NOTA-c(RGDfK); Figure S4. ESI-MS spectrum of ^nat^Cu-NOTA-TP-c(RGDfK); Figure S5. Radio-TLC of the radiolabelling and the plasma stability of [^64^Cu]Cu-NOTA-TP-c(RGDfK. (A) ^64^Cu (free); (B) [^64^Cu]Cu-NOTA-TP-c(RGDfK); (C) [^64^Cu]Cu-NOTA-TP-c(RGDfK after 48 h incubation in plasma; Figure S6. Inhibition of [^64^Cu]Cu-NOTA-c(RGDfK) binding to integrin on U87MG cells with various concentrations of ^nat^Cu-NOTA-c(RGDfK) and ^nat^Cu-NOTA-TP-c(RGDfK); Figure S7. Cytotoxicity of ^64^Cu-NOTA conjugates on U87 MG and SVG p12 normal cells assessed by PrestoBlue assay at 24, 48 and 72 h; Table S1. Uptake kinetic of ^64^Cu-NOTA conjugates on U87 MG and SVG p12 cell line from 1 h to 48 h (*n* = 3); Table S2. Internalized activity of ^64^Cu-NOTA conjugates on U87 MG and SVG p12 cell line from 1 h to 48 h (*n* = 3); Table S3. The efflux rate of ^64^Cu-NOTA conjugates on U87 MG and SVG p12 cell line from 0 to 24 h.

## Abbreviations

The following abbreviations are used in this manuscript:

CH_3_CN	Acetonitrile
CPM	Count per minute
c(RGDfK	Cyclic arginine-glycine-aspartate-D-phenylalanine-lysine
CRT	Chemoradiotheranostic
^64^Cu	Copper-64
^64^Cu(OAc)_2_	^64^Cu-Acetate
^64^CuCl_2_	^64^Cu-Chloride
DCM	Dichlorometane
DIPEA	*N*,*N*-Diisopropylethylamine
DMEM	Dulbecco’s modified Eagle medium
DMF	*N*,*N*-Dimethylformamide
DNA	Deoxyribonucleic acid
EC_50_	Effective concentration
EMEM	Eagle’s minimal essential medium
Equiv	Equivalent
ESI	Electrospray ionization
Fmoc	Fluorenylmethyloxycarbonyl
GBM	Glioblastoma multiforme
H_2_O	Water
HATU	Dimethylamino-*N*,*N*-dimethyl(3H-[1,2,3]triazolo[4,5-b]pyridin-3-yloxy)methaniminium hexafluorophosphate
HOBt	Hydroxybenzotriazole
HPLC	High performance liquid chromatography
HRMS	High-resolution mass spectrometry
IC_50_	Inhibitory concentration
iTLC	Instant thin layer chromatography
ivDde	1-(4,4-Dimethyl-2,6-dioxocyclohex-1-ylidene)-3-methylbutyl
MeOH	Methanol
MG	Malignant glioblastoma
MS	Mass spectrometry
^64^Ni	Nickel-64
NOTA	2,2′,2″-(1,4,7-Triazacyclononane-1,4,7-triyl)triacetic acid
PET	Positron Emission Tomography
Pd(PPh_3_)_4_	Tetrakis(triphenylphosphine)palladium(0)
PhSiH_3_,	Phenylsilane
PyBOP	Benzotriazol-1-yloxytripyrrolidinophosphonium hexafluorophosphate
RGD	Arginine-glycine-aspartate
Rt	Retention time
TACN	Triazacyclononane
TFA	Trifluoroacetic acid
TIPS	Triisopropylsilane
TMZ	Temozolomide
TP	Terpyridine platinum
μM	Micromolar
