# Supplementary material for: A Targeted Radiotheranostic Agent for Glioblastoma: [64Cu]Cu-NOTA-TP-c(RGDfK)
Source: Brain Sci. 2025 Aug 7;15(8):844. doi: 10.3390/brainsci15080844 (PMC12384813; doi:10.3390/brainsci15080844)

SUPPLEMENTAL INFORMATION

A Targeted Radiotheranostic Agent for Glioblastoma: [^{64}Cu]Cu-NOTA-TP-c(RGDfK)

Alireza Mirzaei[1], Samia Ait-Mohand[1], Prenitha Mercy Ignatius Arokia Doss[1], Étienne Rousseau[1,2,3] and Brigitte Guérin[1,2,3*]

Table of content

**Figure S1.** LC/MS/MS spectrum and HPLC chromatogram of NOTA-c(RGDfK) **4**	S2
**Figure S2.** ESI-MS spectrum and HPLC chromatogram of NOTA-TP-c(RGDfK) 5	S2
**Figure S3.** ESI-MS spectrum of ^{nat}Cu-NOTA-c(RGDfK)	S3
**Figure S4.** ESI-MS spectrum of ^{nat}Cu-NOTA-TP-c(RGDfK)	S3
**Figure S5.** Radio-TLC of the radiolabelling and the plasma stability of [^{64}Cu]Cu-NOTA-TP-c(RGDfK). A) ^{64}Cu (free); B) [^{64}Cu]Cu-NOTA-TP-c(RGDfK); C) [^{64}Cu]Cu-NOTA-TP-c(RGDfK) after 48 h incubation in plasma.	S4
**Figure S6.** Inhibition of [^{64}Cu]Cu-NOTA-c(RGDfK) binding to integrin on U87MG cells with various concentrations of ^{nat}Cu-NOTA-c(RGDfK) and ^{nat}Cu-NOTA-TP-c(RGDfK)	S4
**Table S1.** Uptake kinetic of ^{64}Cu-NOTA conjugates on U87 MG and SVG p12 cell line from 1h to 48h (n=3)	S5
**Table S2.** Internalized activity of ^{64}Cu-NOTA conjugates on U87 MG and SVG p12 cell line from 1h to 48h (n=3)	S5
**Table S3.** The efflux rate of ^{64}Cu-NOTA conjugates on U87 MG and SVG p12 cell line from 0 to 24h	S5
**Figure S7.** Cytotoxicity of ^{64}Cu-NOTA conjugates on U87 MG and SVG p12 normal cells assessed by PrestoBlue assay at 24, 48 and 72 h.	S6

Figure S1. LC/MS/MS spectrum and HPLC chromatogram of NOTA-c(RGDfK) **4**

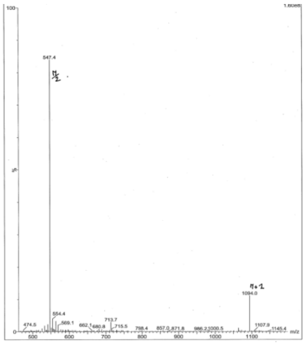

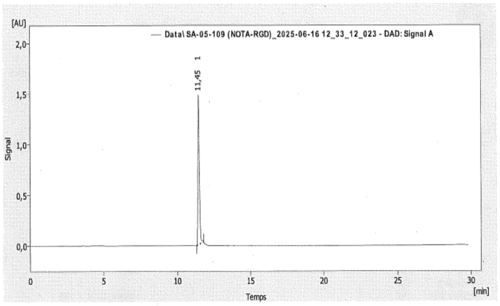

Figure S2. ESI-MS spectrum and HPLC chromatogram of NOTA-TP-c(RGDfK) **5**

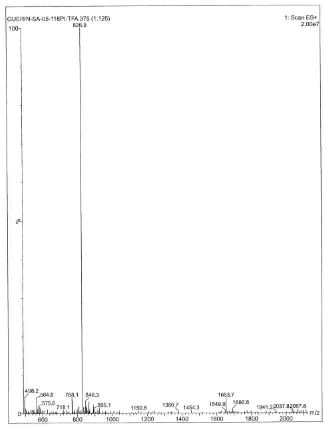

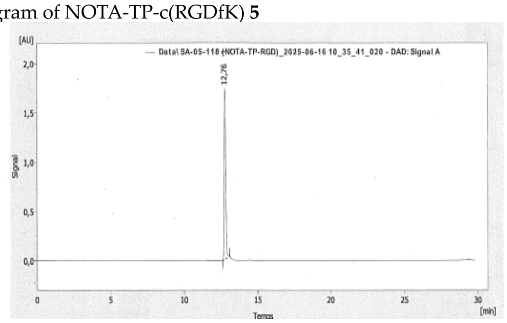

Figure S3. ESI-MS spectrum of natCu-NOTA-c(RGDfK)

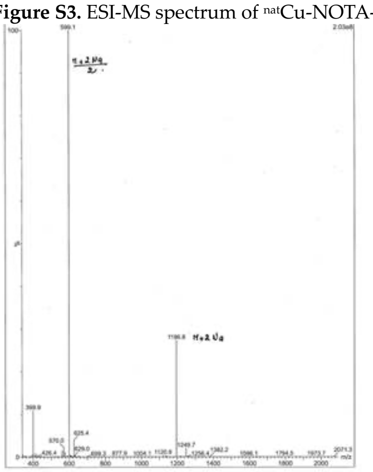

Figure S4. ESI-MS spectrum of natCu-NOTA-TP-c(RGDfK)

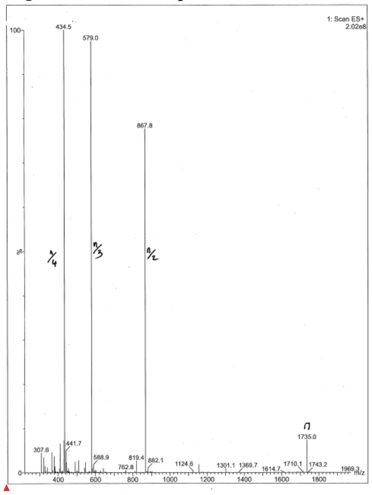

Figure S5. Radio-TLC of the radiolabelling and the plasma stability of [^{64}Cu]Cu-NOTA-TP-c(RGDfK). A) ^{64}Cu (free); B) [^{64}Cu]Cu-NOTA-TP-c(RGDfK); C) [^{64}Cu]Cu-NOTA-TP-c(RGDfK) after 48 h incubation in plasma.

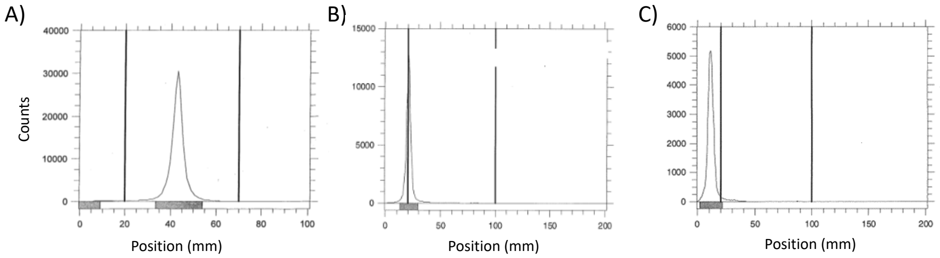

Figure S6. Inhibition of [^{64}Cu]Cu-NOTA-c(RGDfK) binding to integrin on U87MG cells with various concentrations of ^{nat}Cu-NOTA-c(RGDfK) and ^{nat}Cu-NOTA-TP-c(RGDfK)

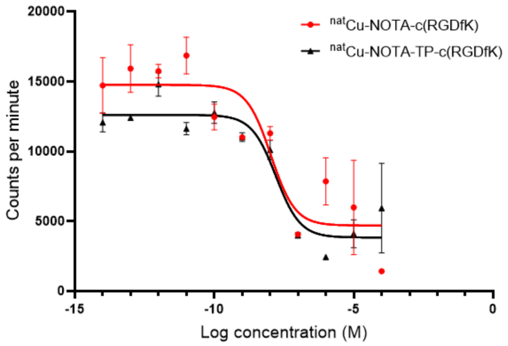

Table S1. Uptake kinetic of ^{64}Cu-NOTA conjugates on U87 MG and SVG p12 cell line from 1h to 48h (n=3)

**^{64}Cu-NOTA conjugates**	**Uptake (% total activity (CPM) /10^{6} cells)**									
	1h		2h		4h		24h		48h	
	U87 MG	SVG p12	U87 MG	SVG p12	U87 MG	SVG p12	U87 MG	SVG p12	U87 MG	SVG p12
[^{64}Cu]Cu-NOTA-TP-c(RGDfK)	6.21 ± 1.58	3.63 ± 0.21	6.89 ±1.73	4.96 ± 0.89	10.33 ± 1.86	5.80 ± 0.49	38.76 ± 1.82	10.87 ± 2.17	37.56 ± 1.30	8.38 ± 1.70
[^{64}Cu]Cu-NOTA-TP-	2.70 ±0.43	2.70 ± 0.58	3.90 ±1.02	3.03 ± 0.68	6.02 ± 2.06	5.28 ± 1.08	30.38 ± 5.53	8.20 ± 1.49	22.35 ±4.70	8.50 ± 0.33
[^{64}Cu]Cu-NOTA-c(RGDfK)	2.50 ±1.00	2.67 ± 0.20	3.23 ±0.61	4.02 ± 0.41	6.08 ± 0.60	5.36 ± 0.94	14.11 ± 0.95	7.90 ± 0.41	18.95 ± 0.45	7.30 ± 0.44

Table S2. Internalized activity of ^{64}Cu-NOTA conjugates on U87 MG and SVG p12 cell line from 1h to 48h (n=3)

**^{64}Cu-NOTA conjugates**	**Uptake (% total activity (CPM) /10^{6} cells)**									
	1h		2h		4h		24h		48h	
	U87 MG	SVG p12	U87 MG	SVG p12	U87 MG	SVG p12	U87 MG	SVG p12	U87 MG	SVG p12
[^{64}Cu]Cu-NOTA-TP-c(RGDfK)	5.50 ± 0.80	2.85 ± 0.56	5.67 ± 0.67	2.46 ± 0.90	7.35 ± 1.31	2.79 ± 0.38	28.03 ± 1.02	7.40 ± 1.43	24.08 ± 1.63	8.21 ± 0.74
[^{64}Cu]Cu-NOTA-TP-	3.19 ± 1.86	1.31 ± 0.11	4.19 ± 2.04	1.64 ± 0.22	6.40 ± 1.83	2.39 ± 0.14	18.41 ± 0.82	7.29 ± 0.80	16.68 ± 1.42	7.98 ± 1.31
[^{64}Cu]Cu-NOTA-c(RGDfK)	3.13 ± 0.31	1.72 ±0.14	3.28 ± 0.26	1.90 ± 0.14	5.00 ± 0.58	3.05 ±0.48	11.44 ± 0.62	6.80 ± 0.32	15.08 ± 0.38	5.98 ± 0.36

Table S3. The efflux rate of ^{64}Cu-NOTA conjugates on U87 MG and SVG p12 cell line from 0 to 24h

**^{64}Cu-NOTA conjugates**	**Uptake (% total activity (CPM) /10^{6} cells)**									
	0.5h		1h		2h		4h		24h	
	U87 MG	SVG p12	U87 MG	SVG p12	U87 MG	SVG p12	U87 MG	SVG p12	U87 MG	SVG p12
[^{64}Cu]Cu-NOTA-TP-c(RGDfK)	88.93 ± 9.47	85.25 ± 8.63	93.32 ± 1.47	75.50 ± 10.93	83.19 ± 4.21	54.29 ± 8.47	76.55 ± 2.59	59.78 ± 2.75	51.36 ± 2.08	31.32 ± 1.01
[^{64}Cu]Cu-NOTA-TP-	97.17 ± 3.04	81.22 ± 3.15	89.38 ± 1.82	76.70 ± 6.30	87.89 ± 1.85	74.32 ± 6.52	71.37 ± 3.71	62.29 ± 3.80	53.23 ± 3.40	28.80 ± 2.81
[^{64}Cu]Cu-NOTA-c(RGDfK)	89.28 ± 6.24	94.55 ± 7.92	85.47 ± 3.64	88.05 ± 6.90	84.08 ± 7.95	83.93 ± 6.98	80.93 ± 6.97	73.44 ± 3.92	67.59 ± 2.23	17.62 ± 1.81

Figure S7. Cytotoxicity of ^{64}Cu-NOTA conjugates on U87 MG and SVG p12 normal cells assessed by PrestoBlue assay at 24, 48 and 72 h

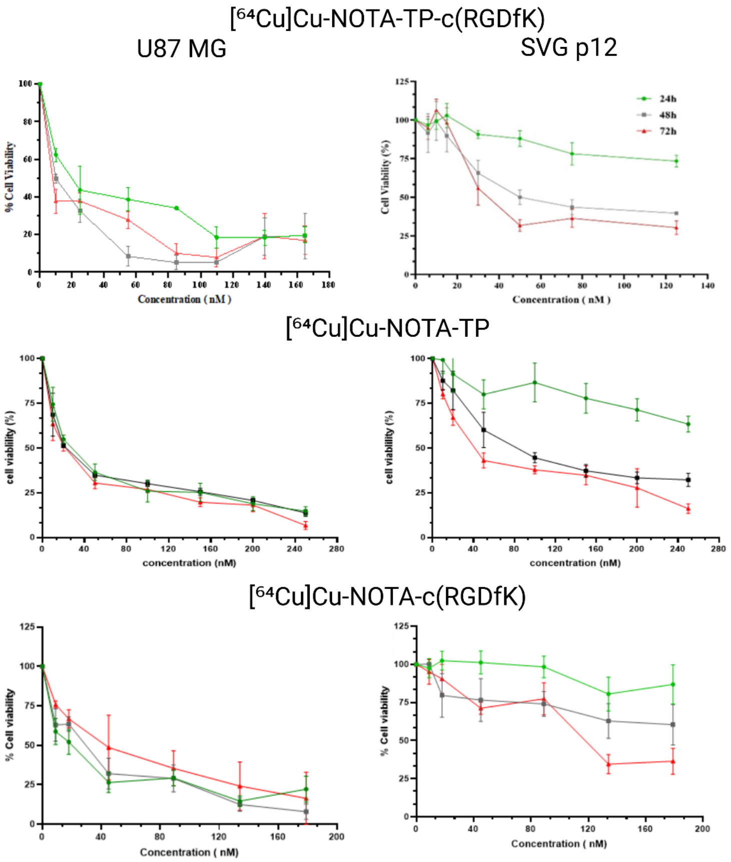

Supplement: Supplementary file 1 [file brainsci-15-00844-s001.zip › brainsci-3760540-supplementary.pdf]
